# How equal is the relationship between individual social capital and psychological distress? A gendered analysis using cross-sectional data from Ghent (Belgium)

**DOI:** 10.1186/1471-2458-14-960

**Published:** 2014-09-16

**Authors:** Veerle Vyncke, Wim Hardyns, Wim Peersman, Lieven Pauwels, Peter Groenewegen, Sara Willems

**Affiliations:** Department of Family Medicine and Primary Health Care, Ghent University, Ghent, Belgium; Research Foundation Flanders, Egmontstraat 5, 1000 Brussels, Belgium; Department of Penal Law and Criminology, Ghent University, Ghent, Belgium; NIVEL, Netherlands Institute for Health Services Research, Utrecht, The Netherlands; Department of Sociology, Department of Human Geography, Utrecht University, Utrecht, The Netherlands

**Keywords:** Social capital, Gender, Psychological distress, Health inequalities

## Abstract

**Background:**

Social capital has been related to various aspects of health. While literature suggests that men and women differently access and mobilize social capital, gender has received little attention within social capital research. This study examines whether the association between individual social capital and psychological distress is different for men and women.

**Methods:**

We made use of data from a representative sample of 1025 adults within 50 neighbourhoods of Ghent (Belgium), collected in the context of the cross-sectional *Social capital and Well-being In Neighbourhoods in Ghent* (SWING) Survey 2011. Six components of social capital were discerned: generalized trust, social support, social influence, social engagement and attachment, the volume of social capital and the mean occupational prestige in one’s network. Multilevel linear regression models were fitted to explore interactions between gender and these components of social capital.

**Results:**

In accordance with previous research, men report lower levels of psychological distress than women (t = 4.40, p < 0.001). Regarding the gender gap in social capital, the findings are mixed. Only for half of the social capital variables (social support, social influence and volume of social capital), a significant gender difference is found, favouring men (t = 4.03, p < 0.001; t = 1.99, p < 0.001 and t = 4.50, p < 0.001 respectively). None of the analysed interaction terms between gender and social capital is significantly related to psychological distress.

**Conclusion:**

The analyses indicate that the association between individual social capital and psychological distress is similar for men and women. The relatively low level of gender stratification in Belgium might have influenced this finding. Furthermore, it is possible that social capital is not of greater importance for women in general, but mainly for women who are in an especially vulnerable social situation that deprives their access to alternative resources (e.g. unemployed women, single mothers). Future studies should seek to identify subgroups for whom social capital might be particularly influential, by transcending ‘simple’ dyads such as ‘men versus women’.

**Electronic supplementary material:**

The online version of this article (doi:10.1186/1471-2458-14-960) contains supplementary material, which is available to authorized users.

## Background

Social capital refers to the idea that social networks are potential resources for individuals, communities, and the society as a whole, and is often used to refer to aspects of social relationships characterized by trust and reciprocity [1, 2]. Social capital has been related to different aspects of individual and population health, such as self-rated health, mental health and health care access [3–6]. Although many authors have reported the beneficial influence of social capital, social capital might also negatively impact health and well-being [7–9]. Strong social bonds within a group might prevent others from joining the network, leading to the exclusion of ‘outsiders’. Furthermore, high levels of social capital can place large claims on group members, due to restricting social control and pressure to conform to the prevailing social norms in the group [7].

Social capital is introduced to the literature by scholars from different scientific disciplines, who each approach the concept from a different theoretical background [10, 11]. Therefore, competing views on the concept can be distinguished in current literature. Most empirical studies on social capital and health have heavily focused on social norms within networks, such as trust and reciprocity, as the core of social capital [12, 13]. However, this focus has been subject to critique since it easily ignores the potential downside of social capital for health and the influence of social stratification on the access to and use of social capital [14, 15]. Consequently, some researchers have proposed a shift in social capital theory from a ‘normative’ to a ‘resource-based’ perspective [16–18]. The latter identifies the resources embedded in social networks as the core of the concept [17] and has some important benefits over the ‘normative’ approach. Due to its strict focus on resources in social networks, this vision enables a clear distinction of social capital from its antecedents and consequences, and facilitates the elaboration of testable hypotheses on social capital and health [15, 16]. The ‘resource-based’ approach to social capital is considered especially useful to study social capital’s role for health inequity since it incorporates the influence of social position on the access to and use of social capital [14, 17].

### Gender differences in social capital

Social capital and, on a related note, social networks are not evenly distributed between both sexes; the social networks of men and women differ in terms of composition, quantity and type. These differences in social capital are believed to arise from gender-specific socialization processes, differential societal expectations with regard to social networking [19] and men and women’s varying opportunities to invest in social capital [20].

Regarding the composition of networks, men generally have less relatives in their networks than women [21, 22]. Furthermore, men are more frequently involved in formal networks [21], while women are traditionally less likely to be involved in relationships that transcend power or authority gradients in society (also referred to as ‘linking social capital’ by Szreter and Woolcock [11]). With regard to the quantity and quality of networks, men generally have larger social networks than women and are more trusting toward others [23]. With regard to the type of networks, women have been found to more frequently participate in community networks [24, 25]. Furthermore, women tend to participate in associations with a caring or domestic focus, or groups associated with education, arts and religion, whereas men mostly participate in associations focused on economy, business, politics or sports [23].

### Gender differences in mental health

Men and women not only differ with regard to their social networks, but also with regard to their health status. More specifically with respect to mental health, the prevalence of most mood disorders and anxiety disorders is higher in women, while most externalizing disorders and substance use disorders are more often found in men [26–28]. With regard to depression, social epidemiologists have consistently reported gender differences, with women reporting more severe and more frequent depressive symptoms [27, 29]. For example, the data from the 2008 Belgian Health Interview Survey shows that 13% of the Belgian women older than 15 report depressive complaints, whereas these complaints are reported by only 6% of their male counterparts [30].

These differences can partially be artefactual, due to for instance differences in reporting depressive symptoms and seeking help. However, literature stresses that differences in mental health between men and women persist after taking these factors into account. The gender gap in mental health can be attributed to different biological, psychological and social factors [26, 27, 31–33]. With regard to biological explanations, it is hypothesized that women are more prone to develop depression due to biochemical mechanisms (e.g. hormonal and neurotransmitter systems) and specific factors related to the reproductive biology of the female body (e.g. menstrual cycle, pregnancy, childbirth and menopause.) [26, 27, 34]. Furthermore, different psycho-social pathways are thought to contribute to gender differences in depression. For instance, personality attributes that predispose to develop depression, such as dependency and self-criticism, are often considered aspects of a female gender identity. Also, the specific social roles women are likely to take up during their lives (e.g. caring and domestic roles, combining work and private life) and the social position of women in society are believed to contribute to the differences in depression rates between men and women [26, 27].

### Can social capital contribute to the explanation of gender differences in mental health

The differential mobilisation of social capital might be one of the psycho-social pathways that underlies the gender gap in mental health problems. However, social capital theory has generally been critiqued to be “gender blind” [35] as the question whether social capital’s association with health differs between men and women has received hardly any attention from the founding fathers of social capital theory [21]. This question is also rarely addressed in empirical research. Empirical articles that address the question whether social capital differently influences men and women’s health are limited and research findings are mixed. Moreover, the few studies that address this research question mostly focus on social capital at the level of local neighbourhoods, to the detriment of research on individual social capital. When gender is found to moderate the association between neighbourhood social capital and health, the positive association of social capital with health is generally stronger for women than for men [24, 25, 36] which could be explained by women’s higher exposure to neighbourhood processes [24, 25, 37].

### Rationale and research aim

The current study explores one of the possible psycho-social pathways that might contribute to gender differences in mental health –social capital- and as such builds upon the current knowledge that gender influences both social network characteristics and aspects of mental health. Although the need for a gendered analysis of social capital’s association with health was stressed in previous research [38], literature has rarely explored whether the association between social capital and health is different for men and women.

Furthermore, current literature has mainly addressed interactions between gender and social capital at the neighbourhood level. As a response to this gap in current knowledge, this study will assess whether the association between individual social capital and psychological distress is similar for men and women.

## Methods

The current study made use of data from the SWING Survey 2011 (N = 1025). The SWING Survey 2011 focused on the role of social processes for disparities in health and well-being at the individual and neighbourhood level. The study protocol was approved by the Ethical Commission of Ghent University and described in detail in the technical report of the SWING-study, which is available at https://biblio.ugent.be/input/download?func=downloadFile&recordOId=4164887&fileOId=4164888.

### Sampling and data collection

The data was collected in Ghent, Belgium. The city covers 158 km^2^ and is divided into 201 ‘statistical sectors’. A statistical sector is the smallest administrative level in which objective administrative data (demographic, social, and economic data) are available and can be compared with the census tract level in the Anglo-Saxon system. In the current study, statistical sectors were used to operationalize neighbourhoods.

Fifty of the 201 neighbourhoods in Ghent were purposely selected by the research team. To be selected, a minimum population size of 200 inhabitants was required to ensure the anonymity of the participants, leaving 142 statistical sectors eligible for selection. Afterwards, a purposive selection of 50 neighbourhoods was made, taking objective data on population density and socioeconomic deprivation [39] into account to reach a representative sample. The inclusion of adjacent neighbourhoods was minimised in order to keep the influence of ‘spatial proximity’ to a minimum. The average population size of the selected neighbourhoods was 1681, while the average size of the selected neighbourhoods was 0.64 km^2^.

In each neighbourhood, a stratified sample of adult inhabitants was selected from the National Population Register, representative of the composition of each neighbourhood with regard to age (18-24, 25-34, 35-44, 45-54, 55-64, 64-74, 75+), sex (male versus female) and nationality (Belgian nationality versus non-Belgian nationality). Table A in the Additional file 1 compares the sample and population characteristics. For each selected inhabitant, three substitutes with similar sociodemographic characteristics were selected. Respondents who could not be reached or refused to participate were replaced by a randomly selected respondent from the same age, gender and ethnic stratum.

A strict data collection protocol, approved by the Ethical Commission of Ghent University Hospital, was followed. The data was collected in the Fall of 2011 by 164 students (2^nd^ bachelor criminological sciences) within the framework of their methodology classes, after receiving an intensive interview training. Data was collected during a home visit using a structured questionnaire that is administered face-to-face. Additionally, respondents were presented some possibly sensitive questions (e.g. questions about income and substance use) in a short self-administered questionnaire. Before being eligible for further analyses, the collected data was put through a strong quality control (e.g. using control respondents, comparing received data with external data with regard to age, nationality and sex, etc.). The overall response rate was 57.13%, but important variations between the different neighbourhoods were observed (neighbourhood level response rates ranged from 36.6 to 76.9%). Table B in the Additional file 1 provides a detailed overview of the response rate per included neighbourhood.

### Dependent variable

Using the 5-item index from the MOS 36-Item Short-Form Health Survey (SF-36), respondents were asked to what extent they had experienced symptoms of depression and nervousness during the past four weeks (referred to as psychological distress throughout the rest of the paper). An overall sum score ranging from 0 to 100 was calculated, with higher scores referring to less psychological distress [40]. A detailed overview of the items that make up this scale and its psychometric characteristics can be found in Table C in the Additional file 1.

### Independent variables

This study includes information on six different components of individual social capital: a scale on *generalized trust* on the one hand and five components which refer to the social resources in respondents’ networks on the other hand. *Generalized trust* is routinely used to measure social capital [41]. In accordance with international research (e.g. European Social Survey), generalized trust was measured using a 3 - item scale.

To measure the other components of social capital, we made use of a ‘Resource Generator’ and a ‘Position Generator’.

A ‘Resource Generator’ [42, 43] lists a number of concrete network resources across different life domains [15]. Respondents were asked to indicate whether someone in their network could provide access to these specific resources. The Resource Generator is considered to be an easily interpretable and valid instrument to measure individual social capital [44] and is to some extent applied in earlier health research [45, 46]. The use of a domain-specific resource generator has been recommended [47], but a Resource Generator developed for health research in the Belgian context was to our knowledge not available. Consequently, a new instrument was developed, based on theory [44, 48] and a Dutch version of the Resource Generator [49].

In line with a theoretical framework on social networks and health [48], three subscales from the resource generator were identified using principal axis factor analysis. As such, three different components of social capital could be distinguished. The subscale on *social support* uses nine items to measure the respondents’ perception of available social support. The subscale on *social influence* consists of three items that refer to the prevailing social norms in one’s network, and asked whether someone in the respondents’ network would encourage them to (1) eat healthy, (2) be physically active and (3) go to a physician if they experience health problems. The subscale on *social engagement and attachment* assessed the sense of fulfilment and closeness respondents ascribe to ties in their social network using 4 items. A detailed overview of the items that were used to measure *generalized trust, social support, social influence and social engagement and attachment*, and the psychometric characteristics of these scales can be found in Table C in the Additional file 1.

In contrast to the Resource Generator, which lists specific health resources in respondents’ networks, the ‘Position Generator’ is a general, non-domain specific instrument to measure individual social capital that focusses on the occupational prestige of alters in the respondents’ network [44]. This instrument listed a number of occupations and asked whether respondents know someone from these specific professions. The occupations were purposely chosen to represent diverse economic disciplines and cover the whole socioeconomic spectrum [44]. The position generator in the current study is based on the 20 item Position Generator used in the Social Cohesion in Flanders (SCIF) study [50], with some minor adjustments based on 11 cognitive interviews used to pretest the instrument. Earlier research has suggested that the Position Generator is a valid and reliable instrument to measure social capital [47] and describes the association between components of individual social capital measured by a Position Generator and different health outcomes [51–54]. Two components of social capital based on the position generator were included in the analyses: *the volume of social capital* on the one hand and *the mean occupational prestige* in one’s network on the other hand. First, the *volume of social capital* was measured by counting the total number of accessed positions [42, 44]. Second, the International Socioeconomic Index of occupational status (ISEI) [55] was used to determine the occupational prestige linked to each of the twenty occupations of the Position Generator. For the categories ‘someone who is on welfare’ and ‘someone who is unemployed’ the occupational prestige scores determined by the researchers of the SCIF-study were used (0 and 5 respectively) [50]. The Position Generator also enabled to calculate the *mean occupational prestige* in respondents’ network. Lin and Dumin state that people’s position on the socioeconomic ladder (as measured by their occupational position) is positively connected with access to social capital [56]. Therefore, having access to social ties with a higher occupational prestige is associated with higher access to social capital and believed to better help individuals to achieve their individual goals [17, 56].

All analyses controlled for age, having a partner (people who currently don’t have a partner were considered as the reference category), experiencing financial difficulties, monthly income per capita and respondents’ sex, by including these sociodemographic variables as covariates in the analyses.

To measure *perceived financial difficulties,* people were asked to report to what extent their household could make ends meet with the available monthly income, using a 5-point Likert scale ranging from ‘very difficult’ to ‘very easy’. *Perceived financial difficulties* was included in the analysis by means of two dummy variables, which discerned between finding it (very) hard to get by (reference category), finding it easy nor hard to get by and finding it (very) easy to get by*.*

Thirteen answering categories were provided to respondents to indicate the total household net income (including wages, salaries, benefits, child support etc.). The monthly *income per capita* (income weighted by household size) was calculated using the OECD modified equivalent scale, based on the mathematical average of the answering categories [57]. Furthermore, all analyses controlled for psychological resilience in an attempt to control for the expected relationship between psychological characteristics and social capital [3]. *Resilience* can be defined as the ability to ‘bounce back’ in times of stress [58, 59]. Research has linked higher levels of resilience to better mental and physical health [60–62]. Comparative research on different instruments to measure resilience has identified the Brief Resilience Scale by Smith and colleagues [59] as a concise, reliable and valid scale to assess resilience. Two items from the Brief Resilience Scale were included in the current study, yielding in a resilience scale with a good internal consistency (see Table C in the Additional file 1 fur further details).

#### Data analysis

Data preparation and descriptive statistics were completed using the Statistical Program for the Social Sciences (SPSS) 21. The differences in psychological distress, social capital and control variables between men and women were explored using T-tests and chi squared tests (see Table 1).

**Table 1:** **Distribution of demographic characteristics, mental health and social capital**

	Total	Men	Women	T/X ^2^
Gender, No. (%)				
*Women*	530 (51.7)			
*Men*	494 (48.2)			
Age, M (sd) (N = 1021)	47 (18.3)	46.14 (17.91)	47.99 (18.72)	t = -1.62
Having a partner, No. (%)				X^2^ = 3.74
*Yes*	779 (76)	389 (78.7)	390 (72.4)	
*No*	246 (24)	105 (21.3)	140 (26.4)	
Having financial difficulties, No. (%)				t = -33.80***
*Very hard to get by*	18 (18)	11 (2.2)	7 (1.3)	
*Hard to get by*	112 (11.1)	35 (7.2)	77 (14.8)	
*Hard nor easy to get by*	289 (28.6)	123 (25.2)	166 (31.9)	
*Easy to get by*	419 (41.4)	211 (43.1)	207 (39.7)	
*Very easy to get by*	173 (17.1)	108 (22.3)	64 (12.3)	
Monthly income per capita, M (sd) (N = 930)	1568.08 (748,37)	1635.18 (760.65)	1506.24 (732.75)	t = -2.63**
Resilience, M (sd) (N = 1022)	7.85 (1.58)	7.97 (1.55)	7.75 (1.61)	t = 2.19**
Generalized trust, M (sd) (N = 1024)	9.86 (2.34)	9.90 (2.40)	9.82 (2.29)	t = 0.50
Social support, M (sd) (N = 1025)	32.23 (13.2)	33.94 (13.30)	30.64 (12.94)	t = 4.03***
Social influence, M (sd) (N = 1025)	9.99 (5.56)	10.34 (5.61)	9.66 (5.51)	t = 1.99**
Social engagement, M (sd) (N = 1024)	20.07 (6.42)	20 (6.52)	20.11 (6.33)	t = -0.236
Volume of social capital, M (sd) (N = 1017)	9.32 (4.63)	10 (4.78)	8.69 (4.41)	t = 4.50***
Mean occupational prestige in network, M (sd) (N = 1006)	47.74 (9.54)	47.54 (9.67)	47.94 (9.44)	t = -0.68
Psychological distress, M (sd) (N = 1021)	74.16 (15.24)	76.30 (14.47)	72.13 (15.67)	t = 4.40***

*p ≤ 0.05, **p ≤ 0.01, ***p ≤ 0.001.

Next, multilevel linear regression models were fitted using maximum likelihood estimation in MLwiN 2.26 to account for the nested data structure of people within neighbourhoods. First, an intercept-only model was fitted to decompose the total variance in psychological distress into the variance of the lowest level errors (individuals) and the highest level errors (neighbourhoods) (see model 0 in Table 2). After examining the neighbourhood-level variance in psychological distress in the intercept-only model, the relationship between individual social capital and psychological distress was analysed, controlling for individual covariates (age, sex, having a partner, financial difficulties and income per capita). These models are fitted for each social capital component separately (see model 1a to model 6a in Tables 2, 3, 4, 5, 6, 7), due to the high correlation between the different components of individual social capital on the one hand (correlation coefficients range up to 0.72 for the association between *social support* and *social influence,* see Table D in the Additional file 1 for further details) and in order to maximize the statistical power of the models on the other hand.

**Table 2:** **Random intercept model (model 0) and multilevel linear model regressing psychological distress on generalized trust, gender and their interaction term**

	Model 0		Model 1a		Model 1b	
	B	95% CI	B	95% CI	B	95% CI
**Fixed effects**						
Constant	74.245	[73.169- 75.321]	68.631	[65.519- 71.743]	68.688	[65.576- 71.800]
Women			-2.862**	[-4.626- -1.098]	-2.864**	[-4.626- -1.102]
Having a partner			1.914	[-0.171- 3.999]	1.875	[-0.210- 3.960]
Easy nor hard to get by			4.092**	[1.134- 7.050]	4.039**	[1.083- 6.995]
(Very) easy to get by			7.349***	[4.389- 10.309]	7.339***	[4.381- 10.297]
Age			0.036	[-0.013- 0.085]	0.037	[-0.012- 0.086]
Income per capita			-0.001	[-0.003- 0.001]	-0.001	[-0.003- 0.001]
Resilience			3.395***	[2.832- 3.958]	3.410***	[2.847- 3.973]
Generalized trust			0.817***	[0.433- 1.201]	0.600***	[0.069- 1.131]
Generalized trust *women					0.441	[-0.304- 1.186]
	**σ** ^**2**^	**SE**	**σ** ^**2**^	**SE**	**σ** ^**2**^	**SE**
**Random effects**						
Level 2 constant	3.645	2.98	0.000	0.000	0.000	0.000
Level 1 constant	228.440	10.36	181.072	8.429	180.808	8.417
Deviance	8456.780		7417.938		7416.595	
Difference in deviance					1.343	
p					0.247	

*p ≤ 0.05, **p ≤ 0.01, ***p ≤ 0.001.

**Table 3:** **Multilevel linear model regressing psychological distress on social support, gender and their interaction term**

	Model 2a		Model 2b	
	B	95% CI	B	95% CI
**Fixed effects**				
Constant	68.589	[65.477-71.701]	68.708	[65.590- 71.826]
Women	-2.564**	[-4.330- -0.798]	-2.574**	[-4.340- -0.808]
Having a partner	1.674	[-0.419-3.767]	1.614	[-0.481- 3.709]
Easy nor hard to get by	4.010**	[1.048-6.972]	3.975**	[1.013- 6.937]
(Very) easy to get by	7.524***	[4.574-10,474]	7.507***	[4.559- 10.455]
Age	0.073	[0,02- 0,124]	0.074**	[0.023- 0.125]
Income per capita	-0.001	[-0,003-0,001]	-0.001	[-0.003- 0.001]
Resilience	3.301***	[2,735-3,867]	3.311***	[2.745- 3.877]
Social support	0.160***	[0,084-0,236]	0.125***	[0.025- 0.225]
Social support *women			0.072	[-0.063- 0.207]
	**σ** ^**2**^	**SE**	**σ** ^**2**^	**SE**
**Random effects**				
Level 2 constant	0.000	0.000	0.000	0.000
Level 1 constant	181.143	8.432	180.928	8.422
Deviance	7418.302		7417.205	
Difference in deviance			1.097	
p			0.295	

*p ≤ 0.05, **p ≤ 0.01, ***p ≤ 0.001.

**Table 4:** **Multilevel linear model regressing psychological distress on social influence, gender and their interaction term**

	Model 3a		Model 3b	
	B	95% CI	B	95% CI
**Fixed effects**				
Constant	67.982	[64.858- 71.106]	68.024	[64.900- 71.148]
Women	-2.755**	[-4.533- -0.977]	-2.756**	[-4.532- -0.980]
Having a partner	1.863	[-0.248- 3.974]	1.838	[-0.275- 3.951]
Easy nor hard to get by	4.542**	[1.571- 7.513]	4.512**	[1.539- 7.485]
(Very) easy to get by	8.238***	[5.290- 11.186]	8.239***	[5.291- 11.187]
Age	0.043	[-0.006- 0.092]	0.043	[-0.006- 0.092]
Income per capita	-0.001	[-0.003- 0.001]	-0.001	[-0.003- 0.001]
Resilience	3.417***	[2.847- 3.987]	3.416***	[2.846- 3.986]
Social influence	0.142	[-0.025- 0.309]	0.086	[-0.143- 0.315]
Social influence *women			0.111	[-0.205- 0.427]
	**σ** ^**2**^	**SE**	**σ** ^**2**^	**SE**
**Random effects**				
Level 2 constant	0.000	0.000	0.000	0.000
Level 1 constant	183.937	8.562	183.842	8.558
Deviance	7432.432		7431.955	
Difference in deviance			0.477	
p			0.490	

*p ≤ 0.05, **p ≤ 0.01, ***p ≤ 0.001.

**Table 5:** **Multilevel linear model regressing psychological distress on social engagement, gender and their interaction term**

	Model 4a		Model 4b	
	B	95% CI	B	95% CI
**Fixed effects**				
Constant	68.887	[65.796- 71.978]	68.895	[65.804- 71.986]
Women	-3.083***	[-4.839- -1.327]	-3.091***	[-4.847- -1.335]
Having a partner	1.569	[-0.509- 3.647]	1.539	[-0.539- 3.617]
Easy nor hard to get by	4.181**	[1.253- 7.109]	4.203**	[1.277- 7.129]
(Very) easy to get by	7.518***	[4.603- 10.433]	7.524***	[4.609- 10.439]
Age	0.050*	[0.001- 0.099]	0.050	[0.001- 0.099]
Income per capita	-0.001	[-0.003- 0.001]	-0.001	[-0.003- 0.001]
Resilience	3.237***	[2.674- 3.800]	3.248***	[2.685- 3.811]
Social engagement	0.399***	[0.256- 0.542]	0.345***	[0.149- 0.541]
Social engagement *women			0.111	[-0.163- 0.385]
	**σ** ^**2**^	**SE**	**σ** ^**2**^	**SE**
**Random effects**				
Level 2 constant	0.000	0.000	0.000	0.000
Level 1 constant	178.382	8.308	178.261	8.308
Deviance	7396.102		7395.480	
Difference in deviance			0.622	
p			0.430	

*p ≤ 0.05, **p ≤ 0.01, ***p ≤ 0.001.

**Table 6:** **Multilevel linear model regressing psychological distress on volume of social capital (number of accessed positions), gender and their interaction term**

	Model 5a		Model 5b	
	B	95% CI	B	95% CI
**Fixed effects**				
Constant	67.489	[64.388- 70.590]	67.601	[64.485- 70.717]
Women	-2.600**	[-4.388- -0.812]	-2.622**	[-4.410- -0.834]
Having a partner	2.176*	[0.077- 4.275]	2.095	[-0.016- 4.206]
Easy nor hard to get by	4.778**	[1.820- 7.736]	4.805**	[1.847- 7.763]
(Very) easy to get by	8.472***	[5.540- 11.404]	8.469***	[5.537- 11.401]
Age	0.039	[-0.014- 0.092]	0.04	[-0.013- 0.093]
Income per capita	-0.001	[-0.003- 0.001]	-0.001	[-0.003- 0.001]
Resilience	3.465***	[2.899- 4.031]	3.462***	[2.896- 4.028]
Volume of social capital	0.030	[-0.182- 0.242]	-0.037	[-0.315- 0.241]
Volume of social capital *women			0.141	[-0.245- 0.527]
	**σ** ^**2**^	**SE**	**σ** ^**2**^	**SE**
**Random effects**				
Level 2 constant	0.000	0.000	0.000	0.000
Level 1 constant	182.236	8.506	182.135	8.501
Deviance	7383.640		7383.130	
Difference in deviance			0.510	
p			0.470	

*p ≤ 0.05, **p ≤ 0.01, ***p ≤ 0.001.

**Table 7:** **Multilevel linear model regressing psychological distress on mean occupational prestige in the network, gender and their interaction term**

	Model 6a		Model 6b	
	B	95% CI	B	95% CI
**Fixed effects**				
Constant	67.708	[64.550- 70.866]	67.715	[64.557- 70.873]
Women	-2.713**	[-4.504- -0.922]	-2.708**	[-4.499- -0.917]
Having a partner	1.992	[-0.137- 4.121]	1.985	[-0.144- 4.114]
Easy nor hard to get by	4.843**	[1.823- 7.863]	4.840**	[1.820- 7.860]
(Very) easy to get by	8.318***	[5.327- 11.309]	8.319***	[5.328- 11.310]
Age	0.036	[-0.013- 0.085]	0.036	[-0.013- 0.085]
Income per capita	-0.001	[-0.003- 0.001]	-0.001	[-0.003- 0.001]
Resilience	3.440***	[2.868- 4.012]	3.441***	[2.869- 4.013]
Mean occupational prestige	0.065	[-0.035- 0.165]	0.084	[-0.057- 0.225]
Mean occupational prestige *women			-0.035	[-0.225- 0.155]
	**σ** ^**2**^	**SE**	**σ** ^**2**^	**SE**
**Random effects**				
Level 2 constant	0.000	0.000	0.000	0.000
Level 1 constant	182.697	8.593	182.672	8.429
Deviance	7273.320		7273.193	
Difference in deviance			0.127	
p			0.722	

*p ≤ 0.05, **p ≤ 0.01, ***p ≤ 0.001.

Next, an interaction term between the component of individual social capital and gender was added to each of the previous models (model 1b to model 6b in Tables 1, 2, 3, 4, 5, 6). The difference in deviance of the nested models with and without the interaction term was analysed to evaluate the importance of the interaction term.

The continuous independent variables (age and resilience) were grand mean centred to simplify the interpretation of the intercept [63].

The item non-response in the current study was very small, ranging from 0.1% to 1.4% for most sociodemographic variables, resilience and psychological distress. Only income per capita showed a higher non-response with 8.7% of the sample not providing data on income level. EM-imputation techniques were used to minimize loss of information in constructing the multiple-item scales. Imputation was only applied when at least half of the questions within a scale construct were answered. The analyses are based on the imputed variables (results using non-imputed scores are virtually identical).

## Results

### Description of the sample

The univariate sample statistics are presented in Table 1. There were 48.2% men and 51.7% women in the sample.

### Distribution of psychological distress and social capital amongst men and women

The mean score for psychological distress was significantly different for men and women (*t* = 4.40; *p* < 0.001); women reported significantly more psychological distress than men (average psychological wellbeing score of 72.13 compared to 76.3 respectively). With regard to half of the social capital components, men reported a significantly higher level of social capital than women. Men reported to have access to more resources via their social network (mean *volume of social capital* = 10) compared to women (mean *volume of social capital* = 8.69, t = 4.50, p < 0.001). Furthermore, they reported a higher number of social ties that might provide *social support* (mean level =33. 94 for men and 30.64 for women, t = 4.03*,* p < 0.001) and more people that encourage healthy behaviour (*social influence*) (mean level =10.34 for men and 9.66 for women, t = 1.99, p < 0.01). There were no significant differences between men and women with regard to levels of *generalized trust*, the *mean occupational prestige* in respondents’ network and levels of *social engagement* ascribed to the network.

### The association between social capital and psychological distress

Tables 2, 3, 4, 5, 6, 7 present the results of the multilevel models. The intercept-only model (model 0) showed that the neighbourhood level accounts for only 1.6% of the total variance in psychological distress. The average psychological distress score of all respondents (N = 923) within all neighbourhoods (N = 50) was 74.25.

Controlling for the effect of background variables (having a partner, monthly income per capita, financial difficulties, gender and age) nullified the variation in psychological distress between neighbourhoods (level 2 variance) (Results not presented, but available upon request). Therefore, further analyses didn’t take neighbourhood-level predictors into account.

After controlling for the background variables and the social capital components, the gender of respondents significantly predicted psychological distress, with men experiencing less psychological distress than women (B = -3.091 to -2.564, *p* < 0.001) (model 1a to model 6a, Tables 2, 3, 4, 5, 6, 7).

Only half of the components of social capital were significantly associated with psychological distress, after the influence of sociodemographic variables and levels of resilience were taken into account (model 1a to model 6a, Tables 2, 3, 4, 5, 6, 7). Being more trusting toward others in general, having higher levels of available supporting ties and feeling more engaged in one’s network were all negatively associated with psychological distress. Having ties that encourage healthy behaviour (*social influence*), the *volume of social capital*, as measured by the total number of accessed positions in the Position Generator, and the *mean occupational prestige* in one’s network did not appear to have a significant influence on psychological distress.

### Gendered analysis of the association between social capital and psychological distress

The interaction term between social capital and gender was not significantly associated with psychological distress in any of the models (B = -0.03 to 0.44, no significant change in the deviance). This suggests that the association between social capital and psychological distress does not significantly differ between men and women.

## Discussion

This study examines whether the association between individual social capital and psychological distress differs for men and women using data from the Social capital and Well-being In Neighbourhoods in Ghent (SWING) Survey 2011.

First of all, the results show that men report significantly less psychological distress, which is in line with earlier national [30] and international research findings on the gender gap in depression rates [26, 28, 29]. Besides, half of the components of social capital are significantly associated with less psychological distress, after controlling for the effect of sociodemographic variables and levels of psychological resilience. Consequently, the results are only partially in line with international research that identifies social capital as a protective determinant of mental health [3, 64]. Regarding the gender gap in social capital, the findings are mixed. Men have higher scores on half of the social capital variables. Men have access to a higher volume of social capital (as measured by the Position Generator) and report a higher number of ties that provide social support and encourage healthy behaviour than women. Research using the World Values Survey found that men are involved in a higher variety of associations [23], which might contribute to their access to a larger set of network resources. Norris & Inglehart further suggest that social and demographic characteristics are associated with factors that enable engagement in social networks. For instance, due to generally more flexible work schedules, more financial possibilities and a less burdensome balance between professional and private life, men are likely in a privileged position for social interaction compared to women [23], which might lead to a different exposure to social networks.

To evaluate whether the association between social capital and psychological distress is different for men and women, interaction terms between gender and social capital are introduced to the analyses. None of the analysed interaction terms are significantly related to psychological distress, which suggests that the association between individual social capital and psychological distress is similar for men and women. Although clear hypotheses on gender differences in the relationship between social capital and health have not been put forward in literature, this is in contrast to what might be expected based on the theory o*f resource multiplication* and *resource substitution* [65]. These theoretical hypotheses are contrasted by Ross & Mirowsky to explore the interaction between gender and education in explaining the gender gap in depression rates*.* According to the theory of resource substitution, the absence of one resource can be counterbalanced by the presence of alternative resources. As a result, the effect of having a specific resource is hypothesized to be greater for those who lack alternative resources. The theory of *resource multiplication* on the other hand states that resources reinforce each other’s impact. Consequently, the presence of a certain resource will have a larger impact on people with more alternative resources, and less impact in the absence of alternative resources [65]. Making the link to different forms of capital, the *resource multiplication* theory would claim that people in an advantaged position on the level of one specific resource (e.g. education, income, social position) will profit to a greater extent from the presence of social capital. This would imply that social capital reinforces existing inequalities in health status and enlarges existing gaps between different social groups. The r*esource substitution* theory on the other hand would suggest that social capital has a higher impact on the life of people who are in a deprived position with respect to other forms of capital, such as people with a low income or a low educational level. The interplay between social capital and other forms of capital is also stressed within the theoretical framework by Bourdieu [66], Lin [17] and other researchers that follow the resource-based approach to social capital [15], although our analyses do not confirm such a dependency.

Different explanations can be sought to explain why the studied interactions between social capital and gender are not significant in the current study. First, it is possible that individual social capital indeed does not play an important role in the existence of gender inequalities in psychological distress in Belgium. Lin & Erickson [67] hypothesize that the differences in social capital between men and women might be relatively small in Western Europe, as these countries are generally less gender-stratified. This might also explain why our findings are in contrast to those of Song [15], who found a significant, yet weak, interaction between gender and individual resources in social networks in relation to depressive symptoms analysing data from the USA, and the observation that our data did not fully support the clear finding of a gender gap in social capital in international literature [15, 68].

### Strengths and weaknesses

A cautious interpretation of the results is recommended, due to some important weaknesses in the study design. The cross-sectional nature of the study makes it difficult to rule out reverse causality, i.e. that people who experience high levels of psychological distress might be more socially isolated and have lower levels of trust [69, 70], especially since social isolation is strongly associated with depression [71]. We have tried to counterbalance this by controlling all analyses for psychological resilience. Furthermore, the response rate ranges within the included neighbourhood ranges from 36.6% to 76.9%. These large differences might partially be explained by important characteristics of neighbourhood inhabitants. For instance, most of the neighbourhoods with the largest response rate are neighbourhoods which are socioeconomically deprived. We know that migrant populations often cluster in these neighbourhoods: an insufficient knowledge of the Dutch language might partially explain the low response rate in these neighbourhoods. Furthermore, our sample might be biased towards people with better than average health and higher levels of social capital, since those people who are the most distrusting and in the worst health status are likely to have declined participation. Consequently, it is possible that the results underestimate the association between social capital and health. The used data also have some important strengths. The data-collection procedure, which combines a face-to-face administration with home visits to pick up the self-administered questionnaires, and the sampling method maximize the inclusion of hard to reach populations (e.g. by providing assistance and clarifications etc.). The extensive operationalization of social capital reflects the multidimensional and complex nature of the concept and is based on validated instruments to measure individual social capital. Finally, it includes components of social capital both following the ‘normative’ (i.e. generalized trust) and ‘resource based’ (based on the Position and Resource Generator) approach to the concept, which is an important contribution to current literature.

## Conclusion

This study indicates that the association between individual social capital and psychological distress is similar for men and women, which is in contrast to what might be expected based on international literature.

Different explanations can be sought to explain why the studied interaction between social capital and gender is not significant.

For instance, it is possible that social capital is not of greater importance for women in general, but that it is more influential for women who are in an especially vulnerable social situation that deprives their access to alternative resources, such as unemployed women, women with a lower educational background and/or single mothers. Single motherhood for instance is associated with a lack of access to individual social capital [72, 73] and hypothesized to contribute to health differences between single and non-single parents [72]. It is as likely that the general distinction between men and women is not able to distinguish those people who are highly at risk of experiencing social exclusion and a lack of resources. Future studies should attempt to identify subgroups in society for whom social capital might be particularly influential, by transcending ‘simple’ dyads such as ‘men versus women’ or ‘singles versus non-singles’.

## Electronic supplementary material

Additional file 1: **Background information with regard to the sample (representativeness and response rate) and the analyses (psychometric properties of independent variables and multicollinearity).** (DOCX 34 KB)

## References

[1] Morrens B (2008). Sociaal kapitaal en gezondheid: een overzicht van de recente onderzoeksliteratuur. Tijdschrift voor Sociologie.

[2] Putnam R (2000). Bowling alone. The collapse and revival of American community.

[3] De Silva MJ, McKenzie K, Harpham T, Huttly SRA (2005). Social capital and mental illness: a systematic review. J Epidemiol Community Health.

[4] Derose KP, Varda DM (2009). Social capital and health care access: a systematic review. Med Care Res Rev.

[5] Kawachi I, Berkman LF, Berkman LF, Kawachi I (2000). Social cohesion, social capital and health. Social epidemiology.

[6] Mansyur C, Amick BC, Harrist RB, Franzini L (2008). Social capital, income inequality, and self-rated health in 45 countries. Soc Sci Med.

[7] Portes A (1998). Social capital: its origins and applications in modern sociology. Annu Rev Sociol.

[8] Ferlander S (2007). The importance of different forms of social capital for health. Acta Sociologica.

[9] Moore S, Daniel M, Gauvin L, Dube L (2009). Not all social capital is good capital. Health Place.

[10] Macinko J, Starfield B (2001). The utility of social capital in research on health determinants. Milbank Q.

[11] Szreter S, Woolcock M (2004). Health by association? social capital, social theory, and the political economy of public health. Int J Epidemiol.

[12] Kawachi I, Subramanian SV, Kim D, Kawachi I, Subramanian SV, Kim D (2008). Social capital and health: A decade of progress and beyond. Social Capital and Health.

[13] Moore S, Haines V, Hawe P, Shiell A (2006). Lost in translation: a genealogy of the “social capital” concept in public health. J Epidemiol Community Health.

[14] Carpiano RM (2007). Neighborhood social capital and adult health: an empirical test of a Bourdieu-based model. Health Place.

[15] Song L (2009). Doctoral thesis. Your body knows who you know: social capital and health inequality.

[16] Carpiano RM (2006). Toward a neighborhood resource-based theory of social capital for health: can Bourdieu and sociology help?. Soc Sci Med.

[17] Lin N (2001). Social capital: a theory of structure and action.

[18] Rostila M (2011). A resource-based theory of social capital for health research: can it help us bridge the individual and collective facets of the concept?. Soc Theor Health.

[19] van Emmerik IIJH (2006). Gender differences in the creation of different types of social capital: a multilevel study. Soc Network.

[20] Leeves GD, Herbert R (2014). Gender differences in social capital investment: theory and evidence. Econ Model.

[21] Ferlander S, Mäkinen IH (2009). Social capital, gender and self-rated health. evidence from the Moscow Health Survey 2004. Soc Sci Med.

[22] Moore G (1990). Structural determinants of men’s and women’s personal networks. Am Sociol Rev.

[23] Norris P, Inglehart R, O’Neill B, Gidengil E (2006). Gendering social capital: Bowling in Women’s Leagues?. Gender and Social Capital.

[24] Chuang Y, Chuang K (2008). Gender differences in relationships between social capital and individual smoking and drinking behavior in Taiwan. Soc Sci Med.

[25] Kavanagh AM, Bentley R, Turrell G, Broom DH, Subramanian SV (2006). Does gender modify associations between self rated health and the social and economic characteristics of local environments?. J Epidemiol Community Health.

[26] Kuehner C (2003). Gender differences in unipolar depression: an update of epidemiological findings and possible explanations. Acta Psychiatr Scand.

[27] Stoppard JM (2000). Understanding depression: feminist social constructionist approaches.

[28] Seedat S, Scott KM, Angermeyer MC, Berglund P, Bromet EJ, Brugha TS, Demyttenaere K, de Girolamo G, Haro JM, Jin R, Karam EG, Kovess-Masfety V, Levinson D, Medina Mora ME, Ono Y, Ormel J, Pennell BE, Posada-Villa J, Sampson NA, Williams D, Kessler RC (2009). Cross-national associations between gender and mental disorders in the World Health Organization World Mental Health Surveys. Arch Gen Psychiatry.

[29] Van de Velde S, Bracke P, Levecque K (2010). Gender differences in depression in 23 European countries. cross-national variation in the gender gap in depression. Soc Sci Med.

[30] Van Der Heyden J, Gisle L, Demarest S, Hesse E, Tafforeau J (2010). Gezondheidsenquête België, 2008. Rapport I: gezondheidstoestand.

[31] Denton M, Prus S, Walters V (2004). Gender differences in health: a Canadian study of the psychosocial, structural and behavioural determinants of health. Soc Sci Med.

[32] Matud MP, Ibáñez I, Bethencourt JM, Marrero R, Carballeira M (2003). Structural gender differences in perceived social support. Pers Indiv Differ.

[33] Piccinelli M, Wilkinson G (2000). Gender differences in depression. Br J Psychiatry.

[34] Halbreich U, Lumleyc LA (1993). The multiple interactional biological processes that might lead to depression and gender differences in its appearance. J Affect Disord.

[35] Gidengil E, O’Neill B, O’Neill B, Gidengil E (2006). Removing Rose Colored Glasses: Examining Theories of Social Capital through a Gendered Lens. Gender and Social Capital.

[36] Stafford M, De Silva M, Stansfeld S, Marmot M (2008). Neighbourhood social capital and common mental disorder: testing the link in a general population sample. Health Place.

[37] Stafford M (2005). Gender differences in the associations between health and neighbourhood environment. Soc Sci Med.

[38] Kawachi I, Berkman LF (2001). Social ties and mental health. J Urban Health.

[39] Vandermotten C, Marissal P, Van Hamme G, Kesteloot C, Slegers K, Vanden Broucke L, Ippersiel B, De Bethune S, Naiken R (2006). Dynamische Analyse van de Buurten in Moeilijkheden in de Belgische stadsgewesten. Grootstedenbeleid.

[40] Ware JE, Sherbourne CD, Davies AR (1988). A short-form general health survey.

[41] Reeskens T, Hooghe M (2008). Cross-cultural measurement equivalence of generalized trust. evidence from the European Social Survey, (2002 and 2004). Soc Indicat Res.

[42] Van der Gaag MPJ, Snijders TAB (2005). The resource generator: social capital quantification with concrete items. Soc Network.

[43] Snijders TAB (1999). Prologue to the measurement of social capital. Tocqueville Rev.

[44] van der Gaag M (2005). The Measurement of Individual Social Capital.

[45] Kobayashi T, Kawachi I, Iwase T, Suzuki E, Takao S (2013). Individual-level social capital and self-rated health in Japan: an application of the Resource Generator. Soc Sci Med.

[46] Webber MP, Huxley PJ (2007). Measuring access to social capital: the validity and reliability of the Resource Generator-UK and its association with common mental disorder. Soc Sci Med.

[47] Van der Gaag MPJ, Snijders TAB, Flap HD, Lin N, Erickson B (2008). Position generator measures and their relationship to other social capital measures. Social capital: advances in research.

[48] Berkman LF, Glass T, Brissette I, Seeman TE (2000). From social integration to health: Durkheim in the new millennium. Soc Sci Med.

[49] Flap HD, Snijders TAB, Völker B, Van der Gaag MPJ (2003). Measurement instruments for social capital of individuals.

[50] Hooghe M, Vanhoutte B, Bircan T (2009). Technical Report for the Social Cohesion Survey Flanders 2009 (SCIF 2009).

[51] Verhaeghe PP, Pattyn E, Bracke P, Verhaeghe M, Van de Putte B (2012). The association between network social capital and self-rated health: pouring old wine in new bottles?. Health Place.

[52] Verhaeghe PP, Tampubolon G (2012). Individual social capital, neighbourhood deprivation, and self-rated health in England. Soc Sci Med.

[53] Song LJ (2011). Social capital and psychological distress. J Health Soc Behav.

[54] Song L, Lin N (2009). Social capital and health inequality: evidence from Taiwan. J Health Soc Behav.

[55] Ganzeboom HBG, De Graaf P, Treiman DJ (1992). A standard international socio-economic index of occupational status. Soc Sci Res.

[56] Lin N, Dumin M (1986). Access to occupations through social ties. Soc Network.

[57] de Vos K, Zaidi A (1997). Equivalence scale sensitivity of poverty statistics for the member states of the European Community. Rev Income Wealth.

[58] Windle G (2010). What is resilience? a review and concept analysis. Rev Clin Gerontol.

[59] Smith BW, Dalen J, Wiggins K, Tooley E, Christopher P, Bernard J (2008). The brief resilience scale: assessing the ability to bounce back. Int J Behav Med.

[60] Davydov DM, Stewart R, Ritchie K, Chaudieu I (2010). Resilience and mental health. Clin Psychol Rev.

[61] Steptoe A, Dockray S, Wardle J (2009). Positive affect and psychobiological processes relevant to health. J Pers.

[62] Eriksson M, Lindström B (2006). Antonovsky’s sense of coherence scale and the relation with health: a systematic review. J Epidemiol Community Health.

[63] Hox J (2002). Multilevel analysis: techniques and applications.

[64] Almedom AM (2005). Social capital and mental health: an interdisciplinary review of primary evidence. Soc Sci Med.

[65] Ross CE, Mirowsky J (2006). Sex differences in the effect of education on depression: resource multiplication or resource substitution?. Soc Sci Med.

[66] Bourdieu P, Richardson J (1986). The forms of capital. Handbook of Theory and Research for the Sociology of Education.

[67] Lin N, Erickson BH, Lin N, Erickson B (2008). Theory, measurement, and the research enterprise on social capital. Social capital An international research program.

[68] Lin N (2001). Inequality in social capital: a research agenda. Social capital: a theory of social structure and action.

[69] De Clercq B, Vyncke V, Hublet A, Elgar FJ, Ravens-Sieberer U, Currie C, Hooghe M, Ieven A, Maes L (2012). Social capital and social inequality in adolescents’ health in 601 Flemish communities: a multilevel analysis. Soc Sci Med.

[70] D’Hombres B, Rocco L, Suhrcke M, McKee M (2010). Does social capital determine health? evidence from eight transition countries. Health Econ.

[71] Wang HX, Mittleman MA, Leineweber C, Orth-Gomer K (2006). Depressive symptoms, social isolation, and progression of coronary artery atherosclerosis: the Stockholm Female Coronary Angiography Study. Psychother Psychosom.

[72] Westin M, Westerling R (2007). Social capital and inequality in health between single and couple parents in Sweden. Scand J Public Health.

[73] Song L (2012). Raising network resources while raising children? access to social capital by parenthood status, gender, and marital status. Soc Network.

[CR74] The pre-publication history for this paper can be accessed here:http://www.biomedcentral.com/1471-2458/14/960/prepub

