# Supplementary material for: How equal is the relationship between individual social capital and psychological distress? A gendered analysis using cross-sectional data from Ghent (Belgium)
Source: BMC Public Health. 2014 Sep 16;14:960. doi: 10.1186/1471-2458-14-960 (PMC4177588; doi:10.1186/1471-2458-14-960)
Supplement: Supplementary file 1 — Additional file 1: Background information with regard to the sample (representativeness and response rate) and the analyses (psychometric properties of independent variables and multicollinearity). (DOCX 34 KB) [file 12889_2013_7078_MOESM1_ESM.docx]

**Additional file 1**

Table A: Comparison of sample and population, with regard to age, sex and nationality.

|  | **Population (%)** | **Sample (%)** |
| --- | --- | --- |
| **Age** |  |  |
| 18-24 | 9.6 | 10.0 |
| 25-34 | 22.2 | 20.7 |
| 35-44 | 18.2 | 18.0 |
| 45-54 | 16.4 | 17.7 |
| 55-64 | 12.8 | 13.4 |
| 65-74 | 9.9 | 10.0 |
| + 75 | 10.9 | 10.2 |
| **Sex** |  |  |
| Men | 48.6 | 48.3 |
| Women | 51.4 | 51.7 |
| **Nationality** |  |  |
| Belgian nationality | 90.3 | 89.4 |
| Not Belgian nationality | 9.7 | 10.6 |

Table B: Response rate per neighbourhood

| **Neighbourhood** | **Response rate (%)** | **Deprived neig~~b~~hbourhood?** |
| --- | --- | --- |
| 1 | 51.9 | no |
| 2 | 48.6 | no |
| 3 | 67.9 | no |
| 4 | 47.7 | yes |
| 5 | 61.5 | no |
| 6 | 47.8 | no |
| 7 | 60.9 | no |
| 8 | 49.4 | yes |
| 9 | 66.7 | no |
| 10 | 62.5 | no |
| 11 | 45.7 | no |
| 12 | 52.6 | no |
| 13 | 62.5 | no |
| 14 | 61.3 | no |
| 15 | 62.1 | no |
| 16 | 41.9 | yes |
| 17 | 65.5 | no |
| 18 | 69.2 | yes |
| 19 | 76.0 | no |
| 20 | 66.7 | no |
| 21 | 57.1 | no |
| 22 | 55.6 | no |
| 23 | 36.6 | yes |
| 24 | 66.7 | no |
| 25 | 50.0 | yes |
| 26 | 47.4 | no |
| 27 | 52.8 | no |
| 28 | 48.4 | no |
| 29 | 55.9 | yes |
| 30 | 46.9 | yes |
| 31 | 76.0 | no |
| 32 | 65.4 | no |
| 33 | 57.1 | no |
| 34 | 62.5 | no |
| 35 | 58.1 | no |
| 36 | 42.7 | yes |
| 37 | 37.3 | yes |
| 38 | 55.6 | no |
| 39 | 60.0 | yes |
| 40 | 72.0 | no |
| 41 | 49.4 | yes |
| 42 | 65.5 | no |
| 43 | 45.7 | no |
| 44 | 46.7 | yes |
| 45 | 38.8 | no |
| 46 | 67.9 | no |
| 47 | 65.5 | no |
| 48 | 76.9 | no |
| 49 | 60.0 | no |
| 50 | 67.9 | no |

Table C: Overview of included ~~scales~~ multi-item measures, including psychometric properties

|  | **Items** | **Factor loading** | **Alpha value** |
| --- | --- | --- | --- |
|  | DEPENDENT VARIABLE |  |  |
| **Psychological distress** | How often during the past 4 weeks… |  | 0.79 |
|  | …have you been a very nervous person? | 0.58 |  |
|  | …have you felt so down in the dumps that nothing could cheer you up? | 0.74 |  |
|  | …have you felt calm and peaceful? | 0.62 |  |
|  | …have you felt downhearted and blue? | 0.70 |  |
|  | …have you been a happy person? | 0.67 |  |
|  |  |  |  |
|  | INDEPENDENT VARIABLES |  |  |
| **Generalized trust** | To what extent do you agree with the following statements? |  | 0.64 |
|  | Most people can be trusted. | 0.80 |  |
|  | Most people would try to take advantage of you. | 0.59 |  |
|  | Most people try to be helpful. | 0.46 |  |
|  |  |  |  |
| **Social support** | How many people from your personal social network (friends, family, or acquaintances) |  | 0.91 |
|  | …can help you to get your mind off things? | 0.69 |  |
|  | … understand your problems? | 0.67 |  |
|  | … would take you to the doctor/hospital when you are too sick to go there yourself? | 0.76 |  |
|  | … would let you move into their house for a week if you temporarily could not stay at your house? | 0.77 |  |
|  | …would help you with a little job you couldn't do without help, e.g. moving heavy furniture in the house? | 0.80 |  |
|  | … would help with daily chores if you were sick? | 0.77 |  |
|  | … would be able to give advice on the invoice if you would wonder why you had to pay as much at the doctor/dentist? | 0.73 |  |
|  | …would be able to give you legal advice (e.g. when you have conflicts with your landlord, your boss, local authorities, etc.)? | 0.60 |  |
|  | … would be able to give advice in case of a conflict within your family? | 0.70 |  |
|  |  |  |  |
| **Social influence** | How many people from your personal social network (friends, family, or acquaintances) |  | 0.85 |
|  | … would encourage you to exercise (e.g. walking, dancing, riding your bike, doing sports)? | 0.81 |  |
|  | … would encourage you to eat healthy? | 0.86 |  |
|  | …would encourage you to go to the doctor if you experience health problems? | 0.77 |  |
|  |  |  |  |
| **Social engagement** | How many people from your personal social network (friends, family, or acquaintances)… ~~can help you to get your mind of things~~ |  | 0.91 |
|  | … do you feel very close to? | 0.78 |  |
|  | … make you feel good (e.g. make you feel you are useful or make you feel that they are glad to know you)? | 0.89 |  |
|  | … make you feel at home with them? | 0.84 |  |
|  | ..make you feel loved? | 0.87 |  |
|  |  |  |  |
| **Resilience** | To what extent do you agree with the following statements? |  | 0.75 |
|  | It does not take me long to recover from a stressful event. | 0.77 |  |
|  | I usually come through difficult times with little trouble. | 0.77 |  |

Table D: Pearson correlation coefficients illustrating the association between the different components of social capital

|  | **Social support** | **Social influence** | **Social engagement** | **Generalized trust** | **Volume of social capital** |
| --- | --- | --- | --- | --- | --- |
| **Social support** | 1 |  |  |  |  |
| **Social influence** | 0.72*** | 1 |  |  |  |
| **Social engagement** | 0.65*** | 0.51*** | 1 |  |  |
| **Generalized trust** | 0.30*** | 0.21*** | 0.29*** | 1 |  |
| **Volume of social capital** | 0.44*** | 0.34*** | 0.29*** | 0.14*** | 1 |
| **Mean occupational prestige** | 0.19*** | 0.18*** | 0.21*** | 0.19*** | 0.05 |

^* p≤0.05 ** p≤0.01 ***p≤0.001^
